# Associations of obesity defined comprehensively by body mass index and body fat percentage with osteopenia

**DOI:** 10.1016/j.clinsp.2025.100674

**Published:** 2025-05-08

**Authors:** Xin Liu, Yan Lou, Zhiyong Chang, Changyuan Gu, Bin Du, Guangquan Sun

**Affiliations:** Department of Orthopedics and Traumatology, Affiliated Hospital of Nanjing University of Chinese Medicine, Jiangsu Province, PR China

**Keywords:** Obesity, Osteopenia, Body mass index, Body fat percentage, NHANES

## Abstract

**Objective:**

To explore the association of obesity comprehensively defined by Body Mass Index (BMI) and Body Fat percentage (BF%) with osteopenia.

**Methods:**

In this cross-sectional study, data of adult men and postmenopausal women aged ≥ 50 years old were obtained from the National Health and Nutrition Examination Surveys (NHANES) database. Weighted logistic regression analysis was conducted to investigate the association of BF% obesity with osteopenia in participants who had different gender and BMI obesity conditions. The association of obesity comprehensively evaluated by BMI and BF% with osteopenia was also explored in the total population and in gender subgroups.

**Results:**

Among 1720 eligible subjects, 1054 had osteopenia. Multivariate analysis suggested that in males, BMI obesity combined with BF% obesity was associated with higher osteopenia odds compared to BMI obesity only (Odds Ratio [OR = 4.01], 95% Confidence Interval [95% CI 1.43‒11.27]). Compared to participants with both BMI and BF% obesity, those with BMI obesity have lower osteopenia odds (OR = 0.46, 95% CI 0.28‒0.76), whereas those with BF% obesity have higher odds of osteopenia (OR = 2.03, 95% CI 1.35‒3.05, *p* = 0.002). In females, compared to BMI obesity combined with BF% obesity, BF% obesity (OR = 3.37, 95% CI 1.47‒7.73) or non-obesity (OR = 2.11, 95% CI 1.18‒3.75) was respectively associated with higher osteopenia odds. In males, BMI obesity was linked to lower osteopenia odds compared to both BMI and BF% obesity (OR = 0.25, 95% CI 0.10‒0.62).

**Conclusions:**

The comprehensively assessed obesity by BMI and BF% may be more meaningful in the evaluation of potential osteopenia risk, as well as further prevention and intervention of osteoporosis.

## Highlights

• Weighted logistic regression examined Body Fat percentage (BF%) obesity associated with osteopenia across gender and Body Mass Index (BMI) levels.
• In females, compared to BMI obesity combined with BF% obesity, BF% obesity or non-obesity was respectively associated with higher osteopenia odds.
• In males, BMI obesity was linked to lower osteopenia odds compared to both BMI and BF% obesity.

## Introduction

Osteoporosis is a most common chronic disease in the middle-aged and elderly population, characterized by low bone mass, bone strength impairment, and increased fragility fracture risk, leading to heavy family and social burdens.^1^ Osteopenia is widely recognized to be the preclinical stage of osteoporosis, with a global prevalence of approximately 40.4%.^2^^,^^3^ Hence, early screening and intervening in osteopenia are significant for reducing the burdens of osteoporosis and related diseases.

The association between obesity and bone health is complicated, and current evidence is inconsistent. Although most previous studies suggested that obesity might be a protective factor related to osteoporosis and fractures,^4^^,^^5^ there is also a demonstration that obesity could not prevent osteoporosis and may even have harmful effects.^6^ Body Mass Index (BMI) is always a classical index used to measure obesity. In fact, one possible explanation for these inconsistent findings is the limitation that the BMI does not distinguish between fat mass and muscle mass of the human body.^7^ Besides, the Body Fat percentage (BF%) is a commonly used indicator that directly reflects the body fat mass. In recent years, researchers have proposed that BF% may be superior to BMI on predicting the risk of multiple diseases.^8^^,^^9^ In a prospective follow-up study, Lin et al.^10^ considered that the comprehensive evaluation of obesity through both BMI and BF% is better than using a single index in evaluating mortality risk in Chronic Kidney Disease (CKD) patients. Nevertheless, no study has explored the association of obesity that was measured comprehensively via the BMI and BF% with osteopenia.

Herein, the present study based on data extracted from the National Health and Nutrition Examination Survey (NHANES) database aims to explore the association of obesity defined by BMI and BF% comprehensively with osteopenia, and to provide information for early screening and intervention in the osteopenia population.

## Materials and methods

### Study subjects

Data in this cross-sectional study were extracted from the NHANES database in 2005‒2006, 2013‒2014, and 2017‒2018. The NHANES survey is conducted by both the Centers for Disease Control and Prevention (CDC) and the National Center for Health Statistics (NCHS), and assesses the United States noninstitutionalized population’s nutritional and health status. This database integrated a comprehensive, multistage stratified probability sample from selected counties, blocks, households, and individuals. The NCHS well-trained professionals conducted interviews at individuals’ homes, and performed extensive physical examinations at the Mobile Exam Centers (MECs). For detailed information on: https://www.cdc.gov/nchs/nhanes/index.htm.

A total of 6414 individuals were initially included. The inclusion criteria were: Adults aged ≥ 50-years; Males or postmenopausal females (postmenopausal status was defined as self-reported cessation of menstruation for ≥ 12-months); Availability of complete data on BMI, Body Fat Percentage (BF%), Bone Mineral Density (BMD), and covariates (sleep duration, marital status, waist circumference, total 25-hydroxyvitamin D, total energy intake, cotinine, and blood pressure).

The exclusion criteria were: BMI < 18.5 kg/m^2^ (to exclude underweight individuals, as low body weight may independently affect bone health through mechanisms unrelated to obesity); Missing data on osteopenia diagnosis or BF%; Missing values in any of the study covariates listed above.

Finally, 1720 of them were eligible for further analysis. This public database has obtained ethical approval from the Institutional Review Board (IRB) of the NCHS. Since information is de-identified, and participants have provided informed consent, the ethical approval has been waived by the IRB of Affiliated Hospital of Nanjing University of Chinese Medicine. This study follows the STROBE Statement.

### Assessment of obesity through BMI and BF%

Calculation of BMI was according to the formula: BMI = weight (kg) ÷ height^2^ (m^2^), and obesity was recognized as BMI of ≥ 30 kg/m^2^ referring to the World Health Organization (WHO) standard.^11^

In NHANES, whole-body dual-energy X-Ray Absorptiometry (DXA) scans were performed at the MEC through the advanced Hologic QDR 4500 A fan beam X-Ray bone densitometer manufactured by Hologic Inc. Before the DXA scans, persons underwent tests of nuclear medicine or radiographic contrast material during last 72 hours or 3 days, or reported a weight exceeding 300 pounds or a height over 6′·5′ were excluded.^12^ The DXA scan data were collected and analyzed by the Hologic Discovery software, version 12.1 after a rigorous quality control process. It can provide precise measurements of body composition in both total and regional, such as BF%. In this study, obesity was defined as BF% ≥ sex-specific median, where the cutoff values for males and females were respectively 28.2% and 41.3%. In addition, subjects were categorized into four groups based on the comprehensive assessment of obesity using BMI and BF%, including BMI obesity + BF% obesity, BMI obesity + BF% non-obesity, BMI non-obesity + BF% obesity, and BMI non-obesity + BF% non-obesity.

### Diagnosis of osteopenia

Bone Mineral Density (BMD) was measured using DXA scans at four skeletal sites: femoral neck, total femur, trochanter, and intertrochanter. In accordance with the World Health Organization (WHO) diagnostic criteria for osteopenia,^14^ the T-score for each site was calculated as follows:

$$\begin{array}{c} T=\frac{Respondent^{\prime }s\;BMD-Mean\;BMD\;of\;the\;reference\;group}{Standard\;deviation\;\left(SD\right)\;of\;the\;reference\;group} \end{array}$$

The reference group comprised healthy adults aged 20–29 years from the NHANES database in 2005–2006, 2013–2014, and 2017–2018 cycles.^13^ Osteopenia was defined as a T-score between −2.5 and −1.0 at any of the measured skeletal sites (femoral neck, total femur, trochanter, or intertrochanter). This approach aligns with the WHO recommendation that osteopenia can be diagnosed based on T-scores from either the femoral neck or lumbar spine.^14^ Although lumbar spine BMD data were available in NHANES, this study prioritized hip-related measurements (femoral neck, total femur, etc.) to minimize potential confounding effects of degenerative changes in the spine common in older adults.

### Variables selection

Variables could be potential confounding factors were also extracted, including age, race, marital status, gender, educational level, smoking, drinking, Poverty Income Ratio (PIR), physical activity, CKD, Cardiovascular Disease (CVD), anemia, anti-osteoporosis therapy, sleep duration, fracture history, waist circumference, height, weight, Systolic Blood Pressure (SBP), Diastolic Blood Pressure (DBP), fasting glucose, total 25-hydroxyvitamin D, cotinine, total energy intake, Calcium (Ca) intake and caffeine intake.

The information on physical activity was collected by the NHANES questionnaire and converted using the following formula: weekly energy expenditure (MET·min/week) = recommended Metabolic Equivalent (MET) × weekly exercise time of corresponding activity (min), and was categorized with the cut-off value 750 MET·min/week. CKD is recognized as the Urinary Albumin to Creatinine Ratio (UACR) of ≥ 30 mg/g and/or estimated Glomerular Filtration Rate (eGFR) < 60 mL/min/1.73 m^2^. The eGFR is calculated as eGFR = 175 × standardized Serum creatinine (Scr) −1.154 × age −0.203 × 1.212 (if black) × 0.742 (if female), in which the units for GFR and Scr are respectively mL/min/1.73 m^2^ of body surface area and mg/dL. Diagnosis of CVDs (including angina, heart attack, heart failure, coronary heart disease, congestive heart failure, stroke, or cardiovascular drug use) was self-reported. Anemia was diagnosed as serum hemoglobin of < 12 g/dL (males) or < 11 g/dL (females) or having taken treatment for anemia in the past 3-months. The NHANES collected dietary intake information through two 24-hour dietary recall surveys. The first one was conducted in person at the MEC, and the second one was through telephone or mail later in 3‒10 days.^15^ The total energy intake was calculated via dietary intake plus dietary supplement.

### Statistical analysis

Data of continuous variables were presented using mean ± standard error (mean ± SE). Student *t*-test was utilized for the comparison of characteristics of participants between the osteopenia group and non-osteopenia group. Data of categorical variables were presented by frequency and constituent ratio [n (%)]. The Chi-Square test (χ^2^) was performed for comparison. In accordance with the NHANES recommendation, the “Full Sample 2 Year MEC Exam Weight (WTMEC2YR)” should be used for combination analysis of three 2-cycle data.

The covariates associated with osteopenia were screened through weighted univariate logistic regression analysis. Weighted multivariate logistic regression was utilized to investigate the association between BF% obesity and osteopenia in different gender and BMI obesity groups, which was evaluated by Odds Ratios (ORs) with 95% Confidence Intervals (95% *CIs*). Multivariate models adjusted for age, race, anemia, anti-osteoporosis therapy, fracture history and waist circumference. Restricted Cubic Spline (RCS) curves were drawn to fit the nonlinear association between BF% and osteopenia. The associations of obesity comprehensively evaluated by BMI and BF% with osteopenia were also explored in the total study population and in gender subgroups. Additionally, the Receiver Operating Character (ROC) curve with Area Under the Curve (AUC) was drawn to explore the predictive value of the comprehensive index on osteopenia. Statistical analyses were conducted with SAS 9.4 software (SAS Institute, Cary, NC, USA).

## Results

### Characteristics of study subjects

**Fig. 1:**
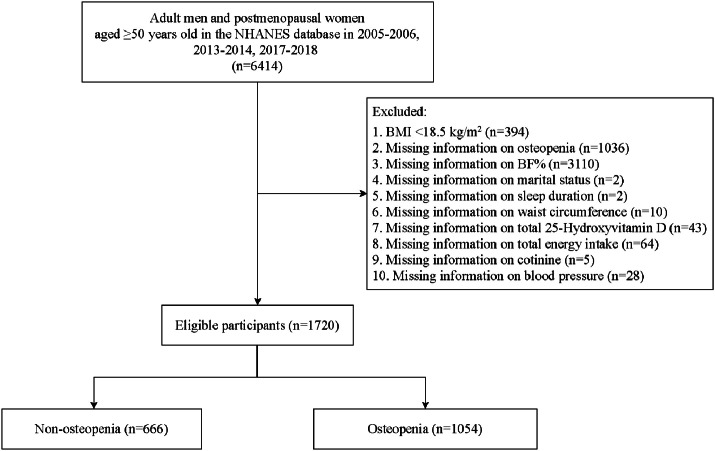
Flow chart of study process.

The study process is shown in Fig. 1. To be specific, 6414 adult men and postmenopausal women aged ≥ 50 years were initially included. Then participants with BMI of < 18.5 kg/m^2^ (*n* = 394), missing information on osteopenia (*n* = 1036), BF% (*n* = 3110), marital status (*n* = 2), sleep duration (*n* = 2), waist circumference (*n* = 10), total 25-Hydroxyvitamin-D (*n* = 43), total energy intake (*n* = 64), cotinine (*n* = 5) and blood pressure (*n* = 28) were excluded 1720 of them were finally eligible.

Among the participants, 1054 had osteopenia. Comparison of characteristics between the non-osteopenia group and osteopenia group was shown in Table 1. More than half of the participants were aged 50‒59 years old (*n* = 1242). Male adults accounted for 61.10% (*n* = 1040). The numbers of individuals with anemia (34 vs. 16), anti-osteoporosis therapy (49 vs. 11), and fracture history (33.87 vs. 23.92) in the osteopenia group were significantly higher than those in the non-osteopenia group. Also, compared to those with non-osteopenia, the median height and weight was significantly lower in osteopenia patients, while the mean waist circumference and cotinine was significantly higher (all *p* < 0.05). 51.56% of participants had BMI obesity in the non-osteopenia group, whereas only 27.85% of participants had BMI obesity in the osteopenia group. Differently, the proportion of BF% obesity in the non-osteopenia group was 56.94%, and that in the osteopenia group was 49.23%.

**Table 1:** Characteristics of participants between non-osteopenia group and osteopenia group.

**Variables**	**Total (*n* = 1720)**	**Non-osteopenia (*n* = 666)**	**Osteopenia (*n* = 1054)**	**Statistics**	**p**
Age, years, n (%)				χ^2^ = 4.169	**0.047**
50‒59	1242 (79.36)	490 (82.34)	752 (77.63)		
60‒69	478 (20.64)	176 (17.66)	302 (22.37)		
Gender, n (%)				χ^2^ = 0.721	0.400
Female	680 (38.90)	266 (37.16)	414 (39.91)		
Male	1040 (61.10)	400 (62.84)	640 (60.09)		
Race, n (%)				χ^2^ = 11.016	**<0.001**
Non-Hispanic white	785 (74.58)	259 (69.20)	526 (77.69)		
Non-Hispanic black	346 (8.42)	197 (12.90)	149 (5.82)		
Mexican American	288 (5.87)	116 (7.08)	172 (5.17)		
Others	301 (11.13)	94 (10.82)	207 (11.32)		
Marital status, n (%)				χ^2^ = 1.681	0.201
Married / living with partner	1132 (69.76)	437 (72.45)	695 (68.21)		
Never married / divorced / separated / widowed	588 (30.24)	229 (27.55)	359 (31.79)		
Educational level, n (%)				χ2 = 0.594	0.550
Less than high school	397 (12.94)	151 (12.15)	246 (13.40)		
High school	410 (26.01)	155 (24.78)	255 (26.72)		
Above high school	913 (61.05)	360 (63.07)	553 (59.88)		
PIR, n (%)				χ^2^ = 1.101	0.347
< 1.3	366 (12.87)	133 (10.74)	233 (14.10)		
1.3‒3.5	575 (30.24)	241 (29.87)	334 (30.45)		
> 3.5	673 (51.76)	262 (55.14)	411 (49.80)		
Unknown	106 (5.13)	30 (4.25)	76 (5.65)		
Smoking, n (%)				χ^2^ = 1.923	0.153
Never	820 (48.76)	335 (51.72)	485 (47.05)		
Former	501 (30.49)	190 (30.97)	311 (30.21)		
Current	399 (20.75)	141 (17.31)	258 (22.74)		
Drinking, n (%)				χ^2^ = 1.202	0.311
No	375 (16.27)	155 (15.53)	220 (16.69)		
Low-to-moderate	898 (59.52)	365 (62.93)	533 (57.55)		
Heavy	135 (9.41)	49 (8.77)	86 (9.78)		
Unknown	312 (14.80)	97 (12.77)	215 (15.98)		
Physical activity, MET·min, n (%)				χ^2^ = 0.030	0.864
< 750	1048 (58.15)	403 (57.77)	645 (58.37)		
≥ 750	672 (41.85)	263 (42.23)	409 (41.63)		
CKD, n (%)				χ² = 0.005	0.942
No	1476 (89.41)	556 (89.51)	920 (89.35)		
Yes	244 (10.59)	110 (10.49)	134 (10.65)		
CVD, n (%)				χ^2^ = 0.651	0.424
No	1406 (83.84)	532 (82.58)	874 (84.57)		
Yes	314 (16.16)	134 (17.42)	180 (15.43)		
Anemia, n (%)				χ^2^ = 7.227	**0.010**
No	1670 (97.73)	650 (98.91)	1020 (97.04)		
Yes	50 (2.27)	16 (1.09)	34 (2.96)		
Anti-osteoporosis therapy, n (%)				χ^2^ = 28.455	**<0.001**
No	1660 (96.46)	655 (99.00)	1005 (94.99)		
Yes	60 (3.54)	11 (1.00)	49 (5.01)		
Sleep duration, hours/d, n (%)				χ^2^ = 0.947	0.385
< 6	240 (12.17)	103 (13.01)	137 (11.68)		
6‒8	1328 (79.68)	518 (80.61)	810 (79.14)		
> 8	152 (8.15)	45 (6.38)	107 (9.18)		
Fracture history, n (%)				χ^2^ = 12.138	**0.001**
No	1000 (69.74)	413 (76.08)	587 (66.13)		
Yes	342 (30.26)	113 (23.92)	229 (33.87)		
Height, cm, Mean ± SE	170.25 ± 0.32	171.47 ± 0.39	169.54 ± 0.42	*t* = −3.598	**0.001**
Weight, kg, Mean ± SE	83.42 ± 0.58	90.65 ± 0.73	79.23 ± 0.75	*t* = −11.479	**<0.001**
Waist circumference, n (%)				χ^2^ = 44.381	**<0.001**
Normal	740 (41.02)	201 (27.27)	539 (48.98)		
High	980 (58.98)	465 (72.73)	515 (51.02)		
SBP, mmHg, Mean ± SE	126.90 ± 0.58	126.66 ± 0.97	127.04 ± 0.65	*t* = 0.350	0.728
DBP, mmHg, Mean ± SE	74.83 ± 0.34	74.97 ± 0.53	74.75 ± 0.41	*t* = −0.341	0.735
Fasting glucose, mg/dL, n (%)				χ^2^ = 2.140	0.125
< 126	687 (41.29)	244 (37.30)	443 (43.60)		
≥ 126	146 (6.22)	67 (6.62)	79 (6.00)		
Unknown	887 (52.49)	355 (56.08)	532 (50.40)		
Total 25-Hydroxyvitamin D, nmoL/L, Mean ± SE	67.85 ± 1.03	66.42 ± 1.62	68.68 ± 1.17	*t* = 1.248	0.218
Cotinine, ng/mL, Mean ± SE	63.77 ± 5.95	52.93 ± 5.79	70.04 ± 7.70	*t* = 2.118	**0.040**
Total energy intake, kcal/d, Mean ± SE	2123.18 ± 25.48	2135.29 ± 48.67	2116.17 ± 40.59	*t* = −0.259	0.796
Ca intake, mg/d, Mean ± SE	927.75 ± 14.13	928.78 ± 24.95	927.16 ± 22.69	*t* = −0.042	0.967
Caffeine intake, mg/d, Mean ± SE	211.38 ± 6.31	196.79 ± 11.53	219.83 ± 7.81	*t* = 1.608	0.115
BMI obesity, n (%)				χ^2^ = 71.474	**<0.001**
No	1097 (63.46)	318 (48.44)	779 (72.15)		
Yes	623 (36.54)	348 (51.56)	275 (27.85)		
BF% obesity, n (%)				χ^2^ = 4.591	**0.038**
No	859 (47.94)	299 (43.06)	560 (50.77)		
Yes	861 (52.06)	367 (56.94)	494 (49.23)		
Comprehensive obesity evaluation, n (%)				χ^2^ = 19.108	**<0.001**
BMI obesity & BF% obesity	499 (29.90)	265 (39.46)	234 (24.38)		
BMI obesity & BF% non-obesity	124 (6.64)	83 (12.10)	41 (3.47)		
BMI non-obesity & BF% obesity	362 (22.15)	102 (17.48)	260 (24.86)		
BMI non-obesity & BF% non-obesity	735 (41.31)	216 (30.96)	519 (47.29)		

*t, t*-test, χ^2^, Chi-Square test.

PIR, Poverty Income Ratio; CKD, Chronic Kidney Disease; CVD, Cardiovascular Disease; SE, Standard Error; SBP, Systolic Blood Pressure; DBP, Diastolic Blood Pressure; Ca, Calcium; BMI, Body Mass Index; BF%, Body Fat percentage.

### Associations of BF% obesity with osteopenia in participants with different gender and BMI obesity conditions

**Fig. 2:**
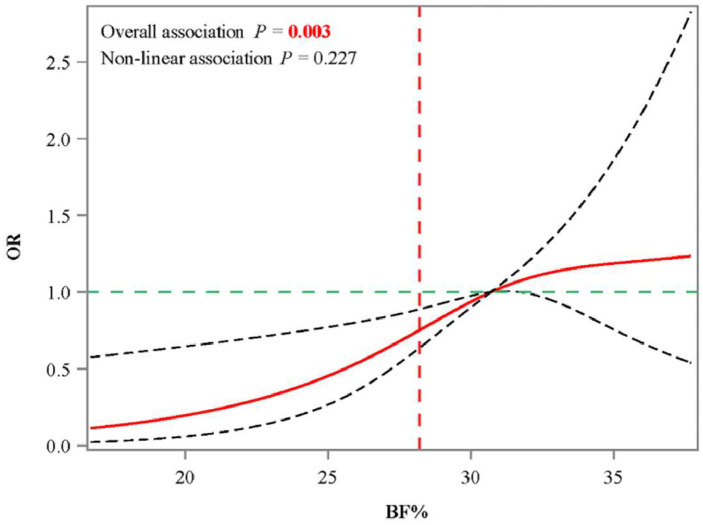
RCS curves of association between BF% and osteopenia in males with BMI obesity. Note: RCS, Restricted Cubic Spline; BF%, Body Fat percentage; BMI, Body Mass Index.

Before exploring the association of BF% obesity with osteopenia in different gender and BMI obesity groups, covariates associated with osteopenia were screened (Supplementary Table 1). The authors observed that age, race, anemia, anti-osteoporosis therapy, fracture history, and waist circumference are significantly linked to the odds of osteopenia (all *p* < 0.05). After adjusting for selected covariates, the authors only observed that in males, having both BMI obesity and BF% obesity was linked to higher odds of osteopenia (OR = 4.01, 95% CI 1.43‒11.27, *p* = 0.011), compared to having BMI obesity only (Table 2). According to the RCS curve of the relationship between BF% and osteopenia odds in men with BMI obesity showed that when BF% > 28.2, the OR values of osteopenia > 1 (Fig. 2).

**Table 2:** Association of BF% obesity with osteopenia in individuals with different gender and BMI obesity conditions.

**Groups**	**BF% obesity (outcome/total)**	**Unadjusted model**		**Adjusted model**^a^	
		**OR (95% CI*)***	**p**	**OR (95% CI)**	**p**
Male & BMI non-obesity	No (313/446)	Ref		Ref	
	Yes (158/235)	0.77 (0.45‒1.32)	0.332	1.60 (0.94‒2.74)	0.082
Male & BMI obesity	No (23/74)	Ref		Ref	
	Yes (146/285)	2.62 (1.08‒6.38)	0.034	4.01 (1.43‒11.27)	**0.011**
Female & BMI non-obesity	No (206/289)	Ref		Ref	
	Yes (102/127)	1.42 (0.73‒2.74)	0.290	1.64 (0.71‒3.77)	0.229
Female & BMI obesity	No (18/50)	Ref		Ref	
	Yes (88/214)	1.51 (0.48‒4.74)	0.467	1.00 (0.24‒4.11)	0.998

BF%, Body Fat percentage; BMI, Body Mass Index; OR, Odds Ratio; CI, Confidence Interval; Ref, Reference.

a Adjusted for age, race, anemia, anti-osteoporosis therapy, fracture history and waist circumference.

### Association of obesity defined comprehensively by BMI and BF% with osteopenia in the total population and in gender subgroups

**Fig. 3:**
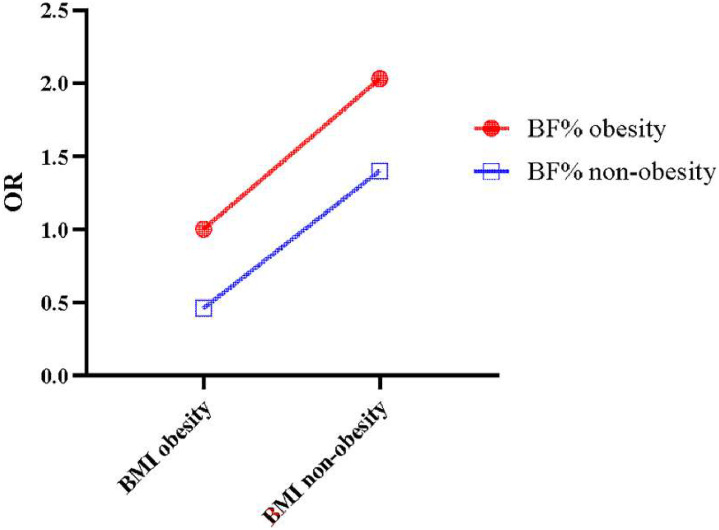
Associations of BMI obesity and BF% obesity with osteopenia respectively in different obesity condition groups. Note: BF%, Body Fat percentage; BMI, Body Mass Index.

The authors further assessed the association of obesity defined by BMI and BF% with osteopenia (Table 3). After adjusting for covariates, comparing to participants with BMI obesity combined with BF% obesity, those with BMI obesity only have lower odds of osteopenia (OR = 0.46, 95% CI 0.28‒0.76, *p* = 0.004), whereas those with BF% obesity only have higher odds (OR = 2.03, 95% CI 1.35‒3.05, *p* = 0.002). Similarly, Fig. 3 clearly shows the association of comprehensively evaluated obesity with osteopenia.

**Table 3:** Association of BMI and BF% compositive defined obesity with osteopenia.

**Variables**	**Unadjusted model**		**Adjusted model**^a^	
	**OR (95% CI)**	**p**	**OR (95% CI)**	**p**
BMI obesity & BF% obesity	Ref		Ref	
BMI obesity & BF% non-obesity	0.47 (0.25‒0.86)	0.017	0.46 (0.28‒0.76)	0.004
BMI non-obesity & BF% obesity	2.30 (1.54‒3.45)	<0.001	2.03 (1.35‒3.05)	0.002
BMI non-obesity & BF% non-obesity	2.47 (1.88‒3.26)	<0.001	1.40 (1.00‒1.97)	0.053

BMI, Body Mass Index; BF%, Body Fat percentage; OR, Odds Ratio; CI, Confidence Interval; Ref, Reference.

a Adjusted for age, race, anemia, anti-osteoporosis therapy, fracture history and waist circumference.

Besides, the association of BMI and BF% comprehensively evaluated obesity with osteopenia was explored in gender subgroups (Table 4). In females, compared to BMI obesity combined with BF% obesity, BF% obesity only (OR = 3.37, 95% CI 1.47‒7.73, *p* = 0.007) or non-obesity (OR = 2.11, 95% CI 1.18‒3.75, *p* = 0.014) was respectively linked to higher odds of osteopenia. Among males, BMI obesity only was linked to lower odds of osteopenia compared to both BMI and BF% obesity (OR = 0.25, 95% CI 0.10‒0.62, *p* = 0.005).

**Table 4:** Association of BMI and BF% compositive defined obesity with osteopenia in gender subgroups.

**Variables**	**Female**		**Male**	
	**OR (95% CI)**	**p**	**OR (95% CI)**	**p**
BMI obesity & BF% obesity	Ref		Ref	
BMI obesity & BF% non-obesity	1.10 (0.29‒4.22)	0.880	0.25 (0.10‒0.62)	0.005
BMI non-obesity & BF% obesity	3.37 (1.47‒7.73)	0.007	1.46 (0.94‒2.27)	0.089
BMI non-obesity & BF% non-obesity	2.11 (1.18‒3.75)	0.014	0.98 (0.62‒1.55)	0.929

BMI, Body Mass Index; BF%, Body Fat percentage; OR, Odds Ratio; CI, Confidence Interval; Ref, Reference.

Adjusted for age, race, anemia, anti-osteoporosis therapy, fracture history and waist circumference.

### Predictive performance of obesity comprehensively calculated by BMI and BF% on osteopenia

**Fig. 4:**
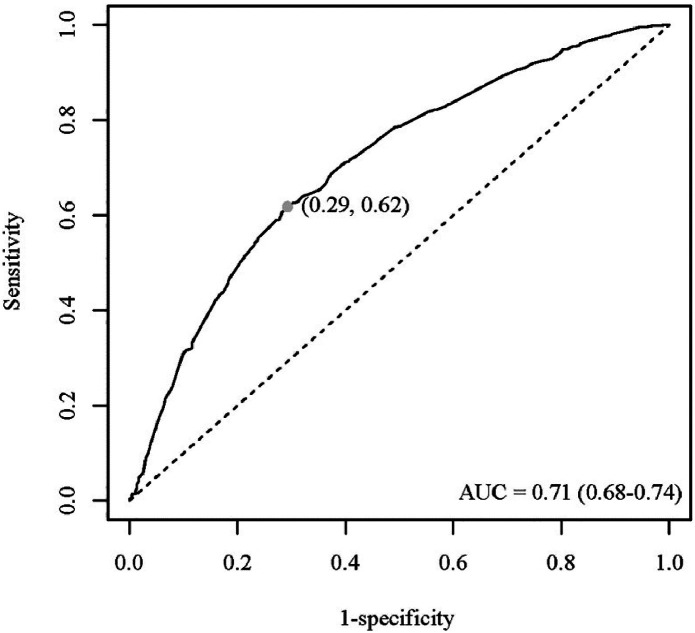
The ROC curve of predictive value of the comprehensive index on osteopenia. Note: ROC, Receiver Operating Character.

Moreover, Fig. 4 shows the ROC curve of the predictive value of the comprehensive index on osteopenia. It clearly suggested that the AUC was 0.71 (0.68‒0.74). Also, the true positive rate of obesity evaluated comprehensively by BMI and BF% was 0.8284, indicating that it had a relatively high sensitivity in predicting osteopenia (Supplementary Table 2).

## Discussion

In the present study, the association of obesity comprehensively assessed by BMI and BF% with osteopenia in adults aged ≥50 years old was investigated based on the NHANES database. The study results suggested that men with both BMI and BF% obesity seemed to have higher odds of osteopenia compared to those with BMI obesity only. Also, comparing with individuals with both BMI and BF% obesity, those who with only BMI obesity have lower odds of osteopenia while those with only BF% obesity have higher odds, after adjusting covariates associated with osteopenia. Moreover, in females, BF% obesity only or non-obesity was respectively associated with higher odds of osteopenia compared to both BMI and BF% obesity.

There is a complex relationship between obesity and bone. Although the broadly accepted notion that obesity has a positive effect on bone health, evidence has challenged this view.^6^^,^^16^ In recent years, the coexistence of osteoporosis and sarcopenia has been considered as a syndrome.^17^ However, due to the BMI is a traditional measurement index for obesity that cannot reflect muscle mass, it may have lower priority than BF% in the assessment of the association between obesity and multiple diseases.^8^^,^^9^ Besides, sarcopenic obesity is a newly identified pathological entity that is characterized by an increased body fat mass with an associated sarcopenia, which was also associated with bone health.^18^ Based on these theoretical foundations, the present study explored the association of obesity measured comprehensively through BMI and BF% with osteopenia, with the aim of providing some new idea for prevention and prevention in the pre-disease stage of osteoporosis. Lin et al.^10^ performed a prospective cohort study that included CKD patients from a medical center in Taiwan and found that BMI-defined obesity was related to significantly lower mortality risk, while the relationship was reversed when obesity was calculated using BF%. The authors similarly observed that persons with BMI obesity have lower odds of osteopenia while those with BF% obesity have higher odds, compared with those who had obesity assessed by both BMI and BF%. Lin et al.^10^ considered this diagnostic discordance may explain the obesity paradox to some extent. Although no study has reported the same topic before, subjects in this study were from the NHANES database, which includes large United States representative populations, so these findings were relatively reliable and may supplement some information on the association between comprehensive measurement of obesity and osteoporosis. However, causal associations of BMI, BF%, and comprehensively measured obesity with bone health need further clarified.

Adipose tissue and bone tissue interact with each other. Adipose tissue plays a role in the musculoskeletal system through a series of substances, including definable hormones such as leptin^19^^,^^20^ and adiponectin.^21^ Besides, obesity as a chronic dysfunction condition with characteristics of a low-grade, systemic inflammatory status. The adiponectin concentration is also inversely proportional to numerous inflammatory cytokines, such as Interleukin (IL)-6, C-Reactive Protein (CRP), and Tumor Necrosis Factor (TNF)-α. Adiponectin can stimulate osteoblastic proliferation, with an increased activity of alkaline phosphatase, as well as the formation of type I collagen and osteocalcin, which are markers of osteoblasts’ differentiation and maturation.^7^ The concentration of adiponectin is usually presented at a low level in obesity.^22^ Herein, it can be presumable that in obesity, chronic inflammatory status can express a high concentration of these inflammation markers, which potently inhibit the expression of adiponectin that may further suppress differentiation and maturation of osteoblasts. The effect of Oxidative Stress (OS) in the development of osteoporosis is also considerable.^23^ OS in obesity, especially sarcopenic obesity, is a crucial factor in muscle function alteration and metabolic dysfunction development.^24^ In addition, a recent study has revealed the interaction between ferroptosis and TNF-α and its impact on osteogenesis and angiogenesis, which might be related to the pathogenesis and regenerative therapy of obesity-related osteoporosis.^25^ Herein, obesity comprehensively measured by both BMI and BF% may partly take full account of the effects of changes in both fat mass and muscle mass. However, due to the observational nature of this study, the mechanism that obesity comprehensively evaluated by both BMI and BF% may more comprehensively reflect the correlation between obesity and osteopenia is still unclear.

The authors further investigated these associations in different gender subgroups. Notably, compared to both BMI and BF% obesity, non-obesity was associated with higher odds of osteopenia in postmenopausal females, which may be related to the phenomenon “obesity paradox”.^26^ However, the potential protective effect of increased BMI and obesity on bone health based on age and gender has not reached a consensus.^7^^,^^27^ In fact, osteoporosis and fracture risk increases along with age, independently from body weight, and, in menopause, the abrupt decrease of estrogen is the primary cause of osteoporosis. Therefore, the authors only included postmenopausal females to avoid this biasing factor. Besides, although obesity measured by BMI and BF% had a positive association with osteopenia risk, it seemed that BF% evaluated obesity played a more negative role. On the contrary, regarding to males, BMI obesity rather than BF% obesity was a potential protective factor for osteopenia, which was consistent with the conclusions of previous studies.^28^^,^^29^

The present findings are further supported by epidemiological evidence highlighting the interplay between obesity and metabolic dysfunction in postmenopausal women. A cross-sectional study of 5027 Brazilian postmenopausal women demonstrated that obesity (BMI > 30 kg/m²) was prevalent in 30% of the cohort and significantly associated with adverse metabolic profiles, including hypertension, elevated triglycerides, fasting glucose, and reduced HDL-C levels.^30^ These metabolic disturbances, such as insulin resistance and dyslipidemia, may exacerbate bone loss by promoting chronic inflammation and altering adipokine secretion (e.g., reduced adiponectin),^31^ potentially explaining the stronger association between BF% obesity and osteopenia observed in the female subgroup. This aligns with the hypothesis that obesity, particularly when characterized by high body fat, reflects a systemic pro-inflammatory state that accelerates bone resorption.

Moreover, the authors have compared multiple influencing factors related to osteopenia between osteopenia patients and non-osteopenia persons, including total energy intake, Ca intake, caffeine intake and total 25-hydroxyvitamin-D concentration.^32^^,^^33^ Unfortunately, no significant difference has been observed. The predictive value of obesity evaluated by both BMI and BF% on osteopenia was assessed, and the AUC of the ROC curve showed a high sensitivity of this comprehensive index. Our results indicated that in addition to the usual recommendation for osteoporosis screening, focusing on comprehensively evaluated obesity may have the potential to be a significant link in osteoporosis prevention. In clinical, it could be an easier and low-cost tool for early screening of populations with high risk of osteopenia. Also, following medical advice to adjust dietary nutrition and take appropriate physical activity to keep both healthy BMI and BF% may be beneficial to reduce the risk of osteopenia and subsequence osteoporosis.

As far as the authors know, this is the first to explore the association of obesity comprehensively evaluated by BMI and BF% with osteopenia in old adults. In this study, BMI and BF% were combined to more accurately distinguish different types of persons with obesity and more comprehensively reflect the correlation between obesity and osteopenia. These results suggested that BF% may be a more valuable indicator of obesity for osteopenia, and provide relevant evidence support for subsequent preventive interventions in osteoporosis. However, there are still some limitations in the present research. Due to the cross-sectional study design, the authors are unable to clarify the causal association of obesity with osteopenia. In addition, the classification cutoff values of BF% were based on the median values of males and females respectively in the study participants, which limited the extrapolation of these results in other populations. Therefore, prospective cohort studies are needed to further validate and supplement the conclusions in the future.

## Conclusion

Obesity evaluated by both BMI and BF% may more comprehensively reflect the association between obesity and osteopenia. In clinical practice, not only BMI but also BF% worth attention in the intervention and prevention of osteoporosis in old adults.

## Declarations

Ethics approval and consent to participate: Not applicable, because NHANES belongs to public databases, the patients involved in the database have obtained ethical approval, users can download relevant data for free for research and publish relevant articles, and the present study is based on open-source data, and the Affiliated Hospital of Nanjing University of Chinese Medicine do not require research using publicly available data to be submitted for review to their ethics committee, so there are no ethical issues and other conflicts of interest.

### Consent for publication

Not applicable, because this paper did not reveal any personal information of patients.

### Availability of data and materials

The datasets used and/or analyzed during the current study were publicly available from the NHANES database.

## Authors’ contributions

Xin Liu, Yan Lou and Guangquan Sun conceived and designed the study.

Xin Liu, Yan Lou, Zhiyong Chang, Changyuan Gu and Bin Du collected the data.

Xin Liu, Yan Lou, Zhiyong Chang, Changyuan Gu and Bin Du analyzed and interpreted the data.

Xin Liu and Yan Lou writing the manuscript.

Guangquan Sun provided critical revisions that are important for the intellectual content.

All authors approve the final version of the manuscript.

## Funding

This study was supported by the 10.13039/501100001809National Natural Science Foundation of China (n° 82,074,471) and the General Foundation of Jiangsu 10.13039/501100010883Province Traditional Chinese Medicine Bureau (n° MS2022020).

## Declaration of competing interest

The authors declare no conflicts of interest.

## Appendix Supplementary materials

Image, application 1
