# Supplementary material for: Associations of obesity defined comprehensively by body mass index and body fat percentage with osteopenia
Source: Clinics (Sao Paulo). 2025 May 8;80:100674. doi: 10.1016/j.clinsp.2025.100674 (PMC12136841; doi:10.1016/j.clinsp.2025.100674)
Supplement: Supplementary file 1 [file mmc1.docx]

**CLINICS-D-25-00044_Supplementary Materials**

**Supplementary Table 1** Covariates associated with osteopenia.

| **Variables** | **OR (95% CI)** | **p** |
| --- | --- | --- |
| Age |  |  |
| 50‒59 | Ref |  |
| 60‒69 | 1.34 (1.00‒1.80) | **0.048** |
| Gender |  |  |
| Female | Ref |  |
| Male | 0.89 (0.68‒1.17) | 0.401 |
| Race |  |  |
| Non-Hispanic white | Ref |  |
| Non-Hispanic black | 0.40 (0.29‒0.55) | **<0.001** |
| Mexican American | 0.65 (0.49‒0.87) | **0.004** |
| Others | 0.93 (0.63‒1.39) | 0.726 |
| Marital status |  |  |
| Married / living with partner | Ref |  |
| Never married / divorced / separated / widowed | 1.23 (0.89‒1.68) | 0.202 |
| Educational level |  |  |
| Less than high school | Ref |  |
| High school | 0.98 (0.65‒1.48) | 0.913 |
| Above high school | 0.86 (0.61‒1.21) | 0.384 |
| PIR |  |  |
| < 1.3 | Ref |  |
| 1.3‒3.5 | 0.78 (0.50‒1.20) | 0.248 |
| > 3.5 | 0.69 (0.44‒1.09) | 0.107 |
| Unknown | 1.01 (0.40‒2.60) | 0.978 |
| Smoking |  |  |
| Never | Ref |  |
| Former | 1.07 (0.78‒1.47) | 0.656 |
| Current | 1.44 (0.99‒2.11) | 0.056 |
| Drinking |  |  |
| No | Ref |  |
| Low-to-moderate | 0.85 (0.58‒1.24) | 0.390 |
| Heavy | 1.04 (0.68‒1.59) | 0.859 |
| Unknown | 1.16 (0.73‒1.87) | 0.521 |
| Physical activity |  |  |
| < 750 | Ref |  |
| ≥ 750 | 0.98 (0.73‒1.30) | 0.864 |
| CKD |  |  |
| No | Ref |  |
| Yes | 1.02 (0.63‒1.64) | 0.942 |
| CVD |  |  |
| No | Ref |  |
| Yes | 0.86 (0.60‒1.24) | 0.424 |
| Anemia |  |  |
| No | Ref |  |
| Yes | 2.76 (1.25‒6.08) | **0.013** |
| Anti-osteoporosis therapy |  |  |
| No | Ref |  |
| Yes | 5.20 (2.60‒10.39) | **<0.001** |
| Sleep duration |  |  |
| < 6 | 0.91 (0.54‒1.56) | 0.738 |
| 6‒8 | Ref |  |
| > 8 | 1.47 (0.86‒2.51) | 0.157 |
| Fracture history |  |  |
| No | Ref |  |
| Yes | 1.63 (1.22‒2.17) | **0.002** |
| Waist circumference |  |  |
| Normal | Ref |  |
| High | 0.39 (0.29‒0.52) | **<0.001** |
| SBP | 1.00 (0.99‒1.01) | 0.728 |
| DBP | 1.00 (0.99‒1.01) | 0.735 |
| Fasting glucose |  |  |
| < 126 | Ref |  |
| ≥ 126 | 0.78 (0.46‒1.30) | 0.326 |
| Unknown | 0.77 (0.58‒1.01) | 0.062 |
| Total 25‒Hydroxyvitamin D | 1.00 (1.00‒1.01) | 0.228 |
| Total energy intake | 1.00 (1.00‒1.00) | 0.796 |
| Ca intake | 1.00 (1.00‒1.00) | 0.967 |
| Caffeine intake | 1.00 (1.00‒1.00) | 0.159 |

OR, Odds Ratio; CI, Confidence Interval; Ref, Reference; PIR, Poverty Income Ratio; CKD, Chronic Kidney Disease; CVD, Cardiovascular Disease; SBP, Systolic Blood Pressure; DBP, Diastolic Blood Pressure; Ca, Calcium.

**Supplementary Table 2** Predictive value of obesity comprehensively calculated by BMI and BF% on osteopenia.

| **Variables** | **Value** |
| --- | --- |
| True positive rate | 0.8284 |
| True negative rate | 0.4144 |
| False positive rate | 0.5856 |
| False negative rate | 0.1716 |

BMI, Body Mass Index; BF%, Body Fat percentage.
